# Microbial metabolite mimicry: one step closer to drug discovery

**DOI:** 10.18632/oncotarget.27591

**Published:** 2020-05-12

**Authors:** Sridhar Mani

**Keywords:** microbial metabolites, inflammation, IBD, mimicry, drugs

In 2014, we proposed a hypothesis implicating the role of microbial indoles [indole propionic acid (IPA) in combination with indoles] in regulating innate immunity in the intestine. The pathway implicated a nuclear receptor protein, the pregnane X receptor (PXR), in regulating intestinal inflammation [1]. In 2017, in an editorial in this journal, we proposed the concept of “endobiotic mimics” as new chemical scaffolds to optimize the discovery of relatively non-toxic drugs targeting intestinal inflammation [2]. The mimics would simulate the binding pharmacophore of the metabolites on the receptor. In 2020, we idealized the proof-of-concept that microbial metabolite mimicry is a viable strategy to improve the chemical repertoire in drug discovery [3].

This editorial serves as a summary of the conceptual advance as well as ongoing and future work on the interactions of microbial metabolites and host receptors. A central thesis buried within this concept is the notion that metabolite-like scaffolds are seen in many successful drugs. Natural metabolites are safe, but their binding affinities are generally weak. Scaffold mimicry could theoretically result in less unwanted side-effects than when using unrelated xenobiotics [4].

In our proof-of-this concept, we synthesized several analogs of indole-IPA based on PXR docking leads, which led to identifying FKK6 as a lead chemical hit in PXR-dependent models of inflammation inhibition [3]. Indoles form the core structures of many drugs in clinical use, but it is heartening to know that more recent drug discovery with similar scaffolds exist for the treatment of inflammatory bowel disease. For example, N,N-Bis(benzimidazolelylpicolinonoyl)piperazine, a structural analog of indoles with a benzoyl bridge, is a specific activator of lanthonine synthetase C-like 2 protein, and a potent inhibitor of intestinal inflammation (IBD) in mice [5], and safe in pre-clinical toxicology (no observed adverse effect level) of doses > 1000 mg/kg [6]. Phase I (II) clinical trials are ongoing in humans.

Our efforts now will be to provide more information on many other metabolite-receptor pathways. The existing FKK compounds will be developed for pre-clinical therapeutic lead discovery – efforts are underway for refining our chemical hits. In parallel, specific formulations would be sufficient consideration for us as we move towards an application in IBD. Localized delivery of drugs could aide in the treatment of IBD, and this could be combined with systemic treatments. Furthermore, these strategies will also limit any unwanted systemic side effects. This is of particular importance with regards to PXR (and other similar xenobiotic receptors), in that, PXR is known to induce drug interaction effects, primarily via liver activation of the receptor. The FKK compounds that activate PXR have limited in vitro and in vivo toxicity; furthermore, they do not activate PXR target genes in the liver (and hepatocytes) to any appreciable extent [3].

Overall, our editorial underscores a method to improve drug discovery. There are likely to be limitations with any discovery process, but we formally propose that microbial metabolite mimicry is a new viable aspect to be considered in drug discovery.
